# Self-learning neural network as a prediction model in non-invasive prenatal testing to detect fetal SNVs

**DOI:** 10.1186/s12967-024-05433-y

**Published:** 2024-07-30

**Authors:** Yiming Qi, Chengbin Hu, Jiexia Yang, Ya Gao, Aihua Yin

**Affiliations:** 1 Prenatal Diagnosis Centre, Guangdong Women and Children Hospital, 521 Xingnan St, Guangzhou, Guangdong 511442 China; 2 Maternal and Children Metabolic-Genetic Key Laboratory, Guangdong, 511442 China; 3 BGI Research, Shenzhen, 518000 China; 4 Guangzhou Key Laboratory of Prenatal Screening and Diagnosis, Guangdong, 511400 China; 5 China National GeneBank, Shenzhen, China

**To the Editor**

Recently, two separate teams reported the sequencing of fetal exome with maternal plasma cell-free DNA (cfDNA) to noninvasively detect *de novo* variants, such as single nucleotide variants (SNVs), copy number variants (CNVs), and chromosome abnormalities. [1, 2] These studies demonstrate the potential of cfDNA deep sequencing in expanding the testing range and accuracy of the present noninvasive prenatal screening (NIPS) service. Importantly, one study used the machine learning-based pipeline to model the fetal fraction and read-depth features to assist in CNV identification. [1] This raises interest in how rapid and helpful artificial intelligence (AI) can be used to detect fetal diseases in NIPS. We previously attempted to noninvasively predict fetal genotypes using a haplotype-based method with cfDNA deep whole-genome-sequencing (WGS) data. [3] To validate the value and endorse the use of AI methods in NIPS, we developed a novel self-learning neural network model and implemented it with cfDNA deep sequencing data to detect fetal SNVs causing monogenic diseases.

We enrolled 10 fetuses diagnosed with monogenic diseases (MDs) presenting structural anomalies (Table S1). Maternal plasma was obtained from 19 to 33 gestational weeks (mean 24.9 weeks). Fetal fraction ranged from 2.6 to 14.7% (mean 8.9%). The sequencing depth of each cfDNA sample ranged from 71.4-149.2x (mean 116.64x), while the parental genomes were sequenced with a mean depth of 55.03x (Table S2). A total of 67 features from cfDNA and parental genome data were utilized as input for logistic modeling, resulting in the selection of 56 parameters for neural network design and training. Cord blood WGS data were used to label the neural network (Table S3, supplementary methods). The study workflow is illustrated in Fig. 1A.

**Fig. 1:**
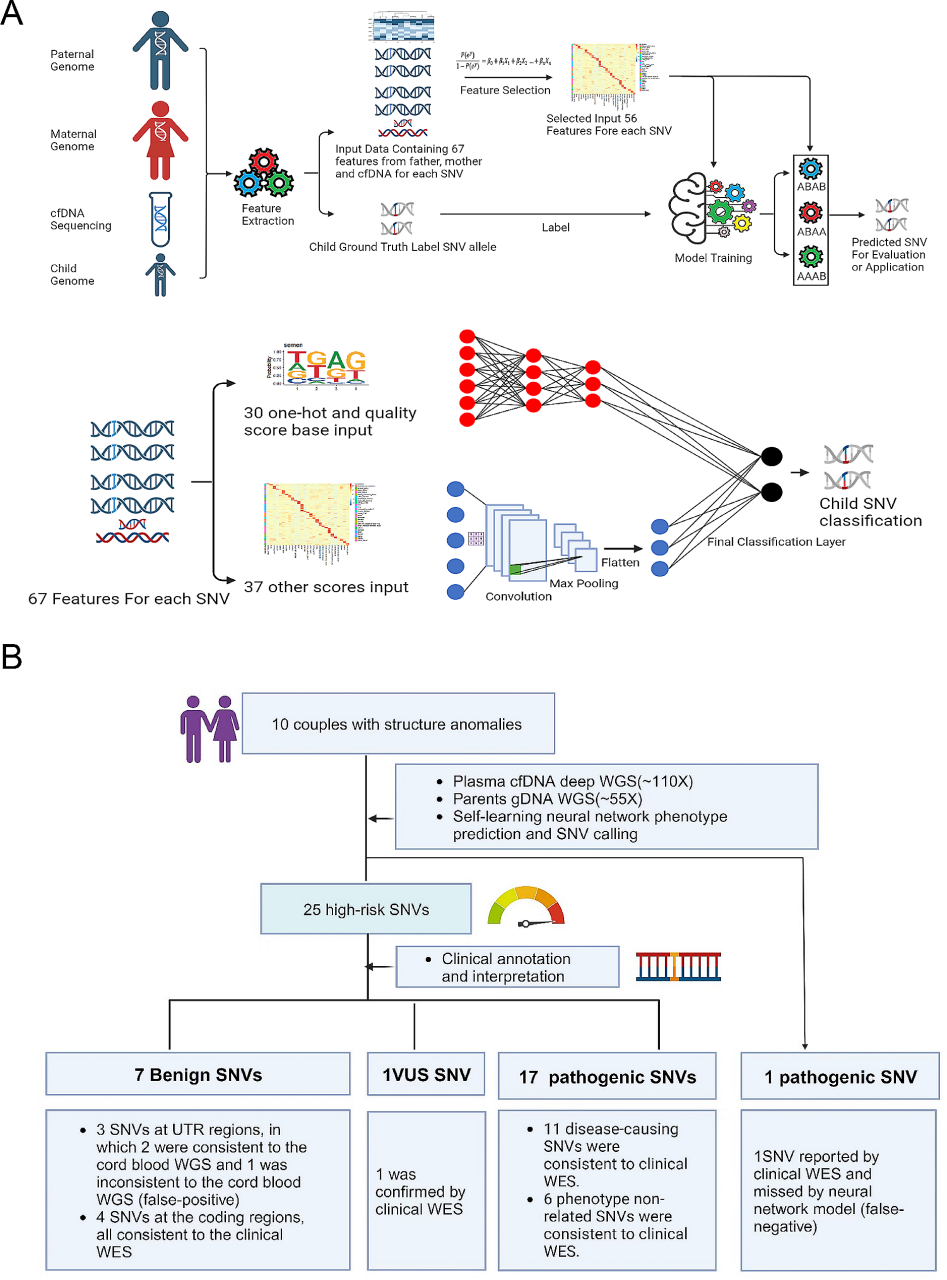
Gives an overview of the workflow (**A**) and the clinical performance (**B**) of the Self-Learning Neural Network model

The self-learning neural network model predicted fetal genotypes with an accuracy ranging from 77.74 to 89.02% compared to cord blood WGS(Table S4). Genotypes were categorized into maternal heterozygous/paternal heterozygous(ABAB), maternal heterozygous/paternal homozygous(ABAA), and maternal homozygous/paternal heterozygous(AAAB) groups, achieving average accuracies of 77.37%, 83.75%, and 95.80%, respectively (Fig. 2). Our deduced fetal genotypes had significantly higher accuracy than previously reported method based on a Bayesian model. [3, 4]

**Fig. 2:**
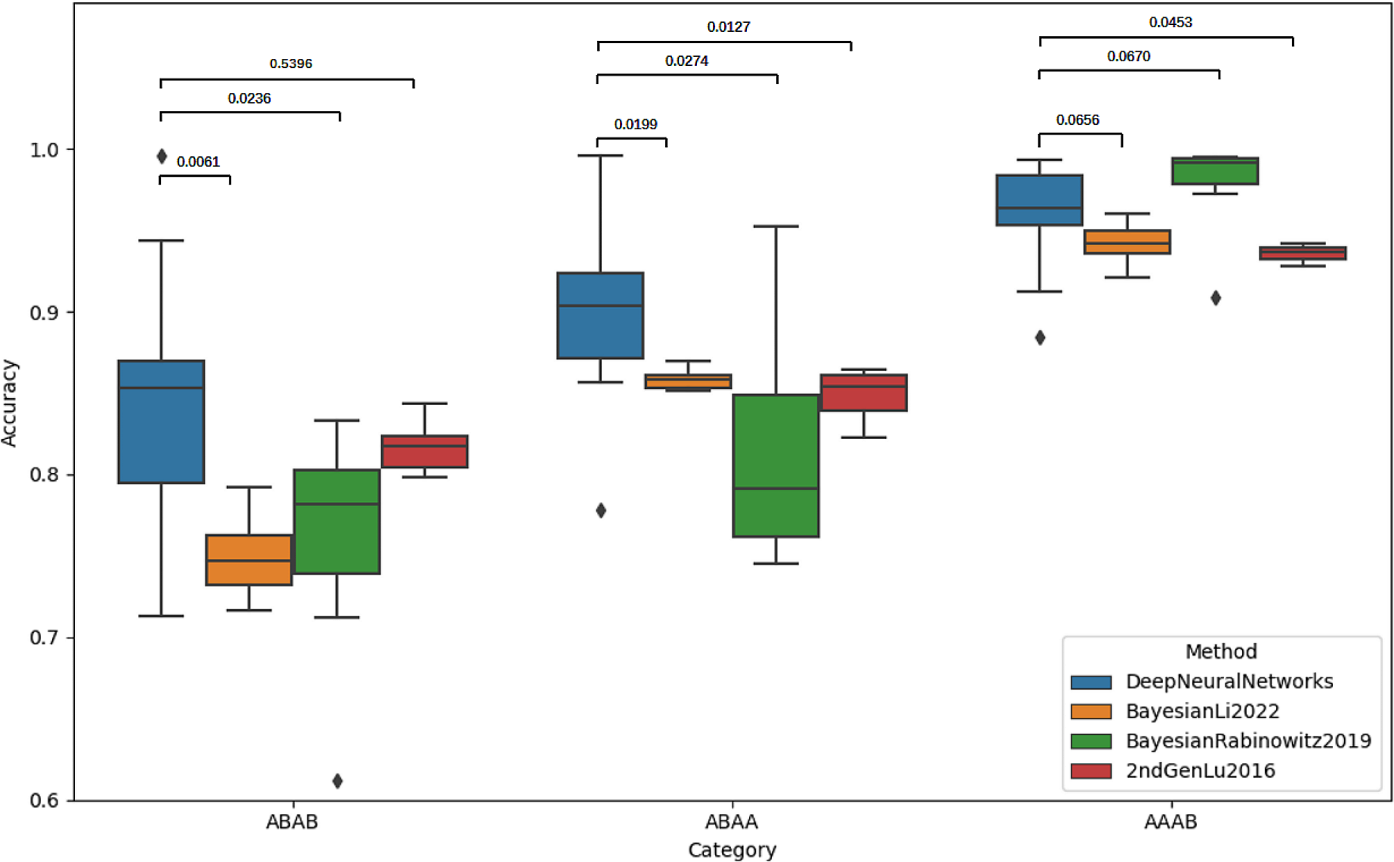
Accuracy comparison across methods and categories Genotypes were categorized into maternal heterozygous/paternal heterozygous (ABAB), maternal heterozygous/paternal homozygous (ABAA), and maternal homozygous/paternal heterozygous (AAAB) groups, Average accuracies of genotype ABAB, ABAA, AAAB were 77.37%, 83.75%, and 95.80%, respectively. Our deduced fetal genotypes (blue) had significantly higher accuracy than previously reported method based on a Bayesian model (orange, green and red)

Using the neural network model, we identified a total of 25 high-risk SNVs in 10 cfDNA samples for further clinical annotation(Supplementary Methods), resulting in 7 benign, 17 pathogenic, and 1 variant of unknown significance (VUS) (Fig. 1B). Among the 25 high-risk SNVs, 22 SNVs of coding regions were confirmed by the clinical whole-exome sequencing (WES) of amniotic fluid, while 3 SNVs at UTR regions were outside the scope of WES and thus validated by cord blood WGS. All 25 high-risk SNVs except one benign SNV at the UTR region of the TOE1 gene had consistent results. The overall sensitivity was 96% (24/25).

In pathogenic SNVs, 11 were associated with clinical phenotypes and determined as disease causing variants, including 2 cases of compound heterozygous recessive diseases, 3 homozygous recessive diseases, 2 hemizygous X-link diseases, and 2 heterozygous dominant diseases (Table S6). All clinical WES reporting SNVs were correctly identified except in SFY22-20, in which a heterozygous SNV causing the dominant disease Loeys-Dietz Syndrome Type 2 could not be detected possibly due to a meager fetal fraction of 2.6%. The overall detection rate was 90% (9/10).

This study demonstrates the robustness of a self-learning neural network in predicting fetal genotypes using deep cfDNA sequencing. Despite the limited sample size, this proof-of-concept study highlights the potential for accurately detecting SNVs associated with monogenic diseases. Future studies with larger cohorts could further enhance the model’s performance and broaden its application to include other genetic variations such as de novo SNVs, indels, CNVs, and chromosomal abnormalities. The promising results indicate that with the rapid development of AI algorithms, this approach could significantly improve the accuracy and scope of non-invasive prenatal testing (NIPT), ultimately benefiting early diagnosis and management of genetic disorders.

### Electronic supplementary material

Below is the link to the electronic supplementary material.

Supplementary Material 1

Supplementary Material 2

## Data Availability

The raw data and detailed methodology can be provided upon request under authors’ consideration.
