# Supplementary material for: Self-learning neural network as a prediction model in non-invasive prenatal testing to detect fetal SNVs
Source: J Transl Med. 2024 Jul 30;22:707. doi: 10.1186/s12967-024-05433-y (PMC11290223; doi:10.1186/s12967-024-05433-y)
Supplement: Supplementary file 1 — Supplementary Material 1 [file 12967_2024_5433_MOESM1_ESM.docx]

Supplementary Appendix

Supplement to:

This appendix has been provided by the authors to give readers additional information about the work.

# Table of contents

[Table of contents 1](#_Toc153899391)

[Methods 2](#_Toc153899392)

[Subject inclusion 2](#_Toc153899393)

[Sample preparation 2](#_Toc153899394)

[DNA Library Construction and Sequencing of DNA Libraries 2](#_Toc153899395)

[Alignment of Sequencing Data 3](#_Toc153899396)

[Neural Network Design and Training 3](#_Toc153899397)

[Clinical diagnosis by WES 6](#_Toc153899398)

[Clinical annotation and interpretation 6](#_Toc153899399)

[Supplementary Tables 8](#_Toc153899400)

[Table S1. Overview of cases included in this study. 9](#_Toc153899401)

[Table S2. Summary of sequencing data in the 10 cases. 1](#_Toc153899402)1

[Table S3. 56 parameters that were used for neural network model design and training using cord blood WGS data. 14](#_Toc153899403)

[Table S4. The precision of deduced fetal genotypes confirmed using cord blood or amniotic fluid WGS 16](#_Toc153899404)

[Table S5. Clinically relevant single nucleotide variants identiﬁed by Self-Learning Neural Network Clinical interpretation of pathogenic/likely pathogenic SNVs determined that was identified high risks and clinical confirmation using WES. 17](#_Toc153899406)

[Table S6. Clinically relevant single nucleotide variants identiﬁed by Self-Learning Neural Network 19](#_Toc153899407)

[References 22](#_Toc153899408)

Methods

Subject inclusion

We recruited 10 families who had been diagnosed with monogenic diseases associated with structure anomalies from our clinical service. This study was approved by the Research Ethics Committee of China Guangdong Women and Children Hospital Clinical (No. 201901091) and the institutional review boards of BGI (BGI-IRB 20002 T3). Informed consent was obtained from all study participants. For each of the ten participants whose plasma DNA samples were sequenced, 5 mL maternal venous peripheral blood was collected in EDTA tubes before their invasive prenatal diagnosis procedures; 5 mL paternal venous peripheral blood in EDTA tubes was also collected. This study also collected leftover samples from the participants who underwent amniocentesis or umbilical cord blood sampling for standard prenatal diagnosis. The results from sequencing data were collected for all cases with available pregnancy outcome data in parallel to neural network modeling.

To be included in the study subjects had to belong to one of the following categories: (a) one or more anomalies/malformations identified in the fetus by ultrasound or MRI examination; (b) increased NT (nuchal translucency) above 3 mm, Intrauterine growth retardation, cystic hygroma or hydrops fetalis; (c) known genetic disorder (parents are carriers of dominant or recessive pathogenic disease).

Sample preparation

Blood samples were centrifuged at 1,600 × g for 10 min at 4 °C. The plasma portion was harvested and re-centrifuged at 16,000 × g for 10 min at 4 °C to remove the remaining cell debris. Cell-free DNA (cfDNA) extraction was performed using the MagPure Circulating DNA KF Kit (Magen), except that 600μL plasma was used for cfDNA extraction. Parental genomic DNA (gDNA) was extracted with the paternal whole blood and maternal buffy coat using the QIAamp DNA Blood Mini Kit (Qiagen). DNA from the Amniotic fluid (AF) and umbilical cord blood (UCB) was extracted with the QIAamp DNA Mini Kit (Qiagen) according to the manufacturer’s tissue protocol.

DNA Library Construction and Sequencing of DNA Libraries

For the ten cfDNA samples subjected to the WGS deep sequencing, DNA libraries were constructed with the Cell-free DNA Library Prep Set (MGI) according to the manufacturer’s protocol, except an adapted PCR amplification reaction condition (98 °C for 2 min, then 10 cycles at 98 °C for 15 s, 56 °C for 15 s, and 72 °C for 30 s and a final extension at 72 °C for 2 min.) for cfDNA library construction. For the parental gDNA, AF gDNA, and UCB gDNA samples, 500ng sonicated DNA was used for library construction. Genomic DNA sequencing libraries were constructed using the Universal DNA Library Prep Set (MGI) following the manufacturer’s protocol. The final DNA library was sequenced on DNBSEQ platforms (MGI, China) using paired-end 100 base pair mode.

Alignment of Sequencing Data

The paired-end sequencing data were analyzed using the SOAP2 in the paired-end mode (20). The paired-end reads were aligned to the nonrepeat-masked reference human genome (hg19). Up to two nucleotide mismatches were allowed for the alignment of each end. To eliminate PCR duplicates, we applied biobambam2 (v2-2.0.87). The genomic coordinates of these potential alignments for the two ends were then analyzed to determine whether any combination would allow the two ends to be aligned to the same chromosome with the correct orientation spanning an insert size with 600 bp and mapping to a single location in the reference human genome for downstream analysis. On average, 98.7% of the reads were aligned to the reference human genome (hg19).

Neural Network Design and Training

Data Preprocess: We obtained 67 features from the sequencing data of plasma cfDNA and parental gDNA, including single nucleotide variants (SNVs), sequencing depth, sequencing quality, chromosomal locations, etc. These became the input for our neural network model to correspond with the fetal genome's alleles at that location. Since each locus has 16 possible combinations (4*4), we established a category of ten distinct allele genotypes, as depicted in Table 1.

Table 1：Allele base pair class

| Base Pair | AA | AT,TA | AC,CA | AG,GA | TT | TC,CT | TG,GT | CC | CG,GC | GG |
| --- | --- | --- | --- | --- | --- | --- | --- | --- | --- | --- |
| Class | 0 | 1 | 2 | 3 | 4 | 5 | 6 | 7 | 8 | 9 |

Feature selection: Before the actual construction of the neural network, we conducted a logistic modeling parameter selection process on the previously generated 67 parameters. We first established an empty logistic model to predict the fetal genotype according to the following Equation 1. We then added each of the 67 parameters to this model and conducted a significance analysis of the final prediction result. From this, we selected the parameters with the most significant differences to construct the first parameter model as shown in Equation 2.

$$\frac{P(e^{y})}{1-P(e^{y})} =\beta_{0} Equation 1$$

$$\frac{P(e^{y})}{1-P(e^{y})} =\beta_{0}+\beta_{1}\times X_{1} Equation 2$$

Following the above steps, we sequentially added the remaining unselected parameters until no parameter could cause a significant change in the parameter model. The finally selected parameters are shown as X1, X2,..., Xn in Equation 3.

$$\frac{P(e^{y})}{1-P(e^{y})} =\beta_{0}+\beta_{1}X_{1}+\beta_{2}X_{2} \ldots+\beta_{n}X_{n} Equation 3$$

Neural network model design and training

After parameter selection, we obtained 56 feature parameters for the established neural network model. We divided the 56 feature parameters into two groups. We focused primarily on the base pair data, as these pairs are crucial in determining the point mutation genotype in offspring. We assigned a binary feature parameter to each type of base, resulting in four distinct feature parameters. For analyzing six base pairs from the father, mother, and cell-free DNA (cfDNA), we utilized a total of 24 feature parameters. Additionally, we incorporated three features to assess whether the genome is homozygous and three quality scores for each locus. With another index feature to show where the SNV located, this brings the total number of feature parameters used in our analysis to 31. The remaining 25 features are quality-related control parameters.We used a fully connected neural network to process the feature parameters of the base sites. Because this type of parameter has the most important impact on the results, we allocated more parameters of this type to the classification layer. In addition, we have 25 remaining quality-related control parameters, including BaseQRankSum, ClippingRankSum, DP, and other quality-related feature parameters. For these parameters, we used a convolutional layer to process them (Equation 4). Since the heterozygosity of the father and mother has a significant influence on the fetal genome, when designing the machine learning model, we model the three different genotypes (ABAB, ABAA, AAAB) separately. This technology does not currently model and predict de novo mutations in the AAAA. Here, we use V1 and V2 to represent two types of feature parameters (V1 for base pair data, V2 for quality-related control parameters). L is the output layer of the fully connected base feature, and W1, W2... Wn are the weight parameter matrices of each layer. Meanwhile, R in equation 5 is the output layer of the quality control parameter convolution. Because we only used one convolutional layer, this convolution and maxpooling only used one layer.

$$L=f_{n}\left( \cdots\left( f_{2}\left( f_{1} \left( V1 \cdot W_{1}+b_{1} \right) \right)\cdot W_{2}+b_{2} \right)+\ldots\right)\cdot W_{n}+b_{n}) Equation 4$$

We first process the feature parameters using a specialized neural network. After this, we merge two components, labeled as L and R, following the structure of our neural network model. Finally, we create a classification model. This model is designed to categorize fetal genomes into 10 different classes. We use data obtained from cord blood sequencing as reference labels for these classes. The final classification model is a simple fully connected neural network with 10 Softmax outputs. The final genotype will be obtained by taking the maximum probability from the 10 Softmax. The final model is represented by Equation 6, where L and R are the results of Equations 4 and 5.

$$R=Maxpool\left( \sum_{j} \sum_{k} filter\left[ j,k \right]V2\left[ m-j,n-k \right] \right) Equation 5$$

$$Result=softmax\left( p_{i} \right)= \frac{e^{p_{i}}}{\sum_{j=1}^{n}e^{p_{j}}}= softmax(\left[ L R \right] \cdot W_{c}+b_{c}) Equation 6$$

The training of the neural network was based on the prior probability distribution of the final result determined by the fetal concentration. We divided the FF into five categories, namely FF<5%, 5%≤FF<10%, 10%≤FF<15%, 15%≤FF<20%, and 20%≤FF. In each FF category, a separate neural network model was trained for different fetal genotypes (ABAB, AAAB, and ABAA). Therefore, Therefore, we have trained a total of 15 neural network models.

Using the neural network model to infer fetal genotypes

To predict the fetal genome, we choose the most suitable model from a pool of 15 based on two key factors: the fetal fraction (FF) and the genetic status (homozygous or heterozygous) at each locus, which can be ABAB, AAAB, or ABAA. For instance, in a case where the FF is 8%, we select and utilize three different models that are designed for the FF range of 5% to less than 10%. The prediction output is then presented as a probability matrix, encompassing all potential genotypes. Under normal circumstances, we consider the genotype with the highest probability in this matrix as the most likely outcome.

Identified high-risk SNVs

After obtaining a highly reliable and high-quality set of variations, use NCBI Clinvar (20230604)

to identify the high-risk variation results. The filtering criteria are as follows: (a) SNP Analysis for Genetic Changes, We scrutinize the single nucleotide polymorphisms (SNPs) to ascertain if they induce alterations in key genetic areas. This includes changes in untranslated regions (UTRs), which are segments of the DNA that do not code for protein but can regulate gene expression; in protein-coding sequences, which are directly responsible for synthesizing proteins; and in amino acids, the building blocks of proteins. Alterations in these areas can have significant implications for protein function and gene regulation. (b) Pathogenicity Assessment, The second crucial step involves determining the pathogenic nature of these variations. Here, we specifically look for evidence that associates the identified SNPs with disease-causing mutations. This involves examining the SNPs' impact on gene function and their known or potential association with genetic disorders or adverse health conditions.

By meticulously following these criteria, we aim to filter out and highlight those genetic variations that pose a significant risk and require further attention for clinical or research purposes.

Clinical diagnosis by WES

Parental blood samples were obtained at the time of the prenatal diagnostic procedure. Subsequently, genomic DNA samples from parent–fetal trios were subjected to WES using an Illumina Nextera Rapid Capture Exome Kit (Illumina, Inc., San Diego, CA, USA) for library preparation. Sequencing was performed with Illumina NovaSeq 5000 (Illumina, Inc., San Diego, CA, USA) with 150-bp paired-end reads, yielding an average coverage above 100×, with 95% of target bases covering at least 20×. The sequencing data regarding the human assembly GRCh37/hgl9 was analyzed by NextGENe software (2.4.1), which is responsible for variation alignment, calls, and annotation. The annotation variants were screened according to the minor allele frequency (MAF) value < 0.01. For the filtered data, genotype-driven variant analysis was conducted combined with phenotype-driven analysis to find the pathogenic or likely pathogenic variants associated with the phenotype. Necessary methodological validation was performed according to different variant types, such as Sanger sequencing, gap-PCR, or qPCR. The database used for variant annotation includes gnomAD (<https://gnomad.broadinstitute.org/>) and 1000 genomes (<https://www.internationalgenome.org/>).

Clinical annotation and interpretation

The sequencing result of the clinical WES and the high-risk SNVs of the neural network model underwent further clinical annotation and interpretation by experienced clinical staff. Low-quality and synonymous variants were firstly filtered out. Variants of specific loci in a pre-established in-house whitelist were retained, including the hotspot mutations, high-frequency loci, splicing and conservative loci, and Pathogenic/Likely Pathogenic variants in the ClinGen, ClinVar, and HGMD database. For mutations not included in the above databases, the Intervar judgment results should be appropriately referred to. Next, the Benign/Likely Benign variants in the ClinGen, ClinVar, HGMD and the variants with population frequency ≤0.005 in 1000G, ESP6500, ExAC, gnomAD_gnome, and a Chinese population frequency database (<https://diseasedx.virgilbio.com/>) were filtered out. Then, the remaining potential Benign/Likely Benign variants were further filter out according to the B/LB and Review status/Submissions/Last evaluated information of the ClinVar database (combined with the B/LB and specific scoring items of Intervar). Lastly, we used both the Exomiser software and our self-developed annotation system to match and score the phenotypes of mutation sites.

Supplementary Tables

Table S1. Overview of cases included in this study.

| Case ID | Maternal Age (years) | GA (weeks) | Fetal Gender | Clinical Phenotypes | Clinical confirmation of disease SNVs | Confirmation of deduced fetal genotypes | Pregnancy Outcome |
| --- | --- | --- | --- | --- | --- | --- | --- |
| SFY22-2 | 26 | 21 | MALE | Ventriculomegaly, echogenic bowel | Clinical WES using UCB | WGS using UCB | TOP |
| SFY22-6 | 30 | 27 | MALE | Hydrocephalus (lateral ventricle:18.8mm/21mm) | Clinical WES using UCB | WGS using UCB | TOP |
| SFY22-8 | 33 | 21 | MALE | Genital malformation, short femur length(-2~3SD) | Clinical WES using AF | WGS using AF | TOP |
| SFY22-9 | 35 | 30 | MALE | Space-occupying in frontal lobes and basal ganglia, accompanied by bleeding, bilateral ventriculomegaly | Clinical WES using AF | WGS using AF | TOP |
| SFY22-13 | 32 | 21 | MALE | Increased nuchal translucency(4.4mm) | Clinical WES using UCB | WGS using UCB | TOP |
| SFY22-14 | 25 | 28 | MALE | Overgrowth of fetal HC and FL(+2SD), Hyperechogenic kidneys | Clinical WES using UCB | WGS using UCB | TOP |
| SFY22-15 | 28 | 22 | MALE | Increased nuchal translucency(7.0mm); Intrauterine growth retardation. | Clinical WES using UCB | WGS using UCB | TOP |
| SFY22-18 | 23 | 33 | MALE | Tetralogy of Fallot, right aortic arch with cervical origin of the left subclavian artery | Clinical WES using AF | WGS using AF | TOP |
| SFY22-19 | 26 | 27 | FEMALE | Increased nuchal translucency(3.5mm) | Clinical WES using UCB | WGS using UCB | TOP |
| SFY22-20 | 29 | 19 | MALE | Multiple fetal abnormalities: strawberry head, distended jugular lymphatic sacs, unilateral multicystic kidney dysplasia | Clinical WES using UCB | WES using UCB | TOP |

GA – gestational age; NT – nuchal translucency; CVS - chorionic villus sample; AF - amniotic fluid; UCB- Umbilical cord blood; WES - whole exome sequencing; WGS - whole genome sequencing; TOP-Termination of pregnancy.

Table S2. Summary of sequencing data in the 10 cases.

| Case No. | Data types | Number of raw reads | Number of mapped reads | Mapping rate | Average depth |
| --- | --- | --- | --- | --- | --- |
| SFY22-2 | Maternal plasma cfDNA deep WGS | 6795812032 | 6740370258 | 99.18% | 124.18 |
| SFY22-2 | Maternal buffy coat WGS | 1494392412 | 1486442329 | 99.47% | 45.50 |
| SFY22-2 | Paternal whole blood WGS | 1132452911 | 1125369587 | 99.37% | 34.42 |
| SFY22-2 | Cord blood WGS | 1280452307 | 1266751467 | 98.93% | 40.41 |
| SFY22-6 | Maternal plasma cfDNA deep WGS | 6538497164 | 6476959678 | 99.06% | 113.75 |
| SFY22-6 | Maternal buffy coat WGS | 2218486870 | 2208031956 | 99.53% | 66.66 |
| SFY22-6 | Paternal whole blood WGS | 2232387115 | 2221306738 | 99.50% | 67.30 |
| SFY22-6 | Amniotic fluid WGS | 1651390496 | 1632729783 | 98.87% | 52.12 |
| SFY22-8 | Maternal plasma cfDNA deep WGS | 6914440064 | 6829549430 | 98.77% | 71.37 |
| SFY22-8 | Maternal buffy coat WGS | 2204871168 | 2193933616 | 99.50% | 66.28 |
| SFY22-8 | Paternal whole blood WGS | 2232387115 | 2221306738 | 99.50% | 67.30 |
| SFY22-8 | Cord blood WGS | 179880465 | 162360108 | 90.26% | 5.06 |
| SFY22-9 | Maternal plasma cfDNA deep WGS | 6265721903 | 6221234405 | 99.29% | 101.18 |
| SFY22-9 | Maternal buffy coat WGS | 2315396577 | 2259407450 | 97.58% | 69.29 |
| SFY22-9 | Paternal whole blood WGS | 2315396577 | 2259407450 | 97.58% | 69.29 |
| SFY22-9 | Amniotic fluid WGS | 1084279438 | 1069750094 | 98.66% | 33.86 |
| SFY22-13 | Maternal plasma cfDNA deep WGS | 4303586957 | 4279486870 | 99.44% | 97.40 |
| SFY22-13 | Maternal buffy coat WGS | 1631117258 | 1624088384 | 99.57% | 49.19 |
| SFY22-13 | Paternal whole blood WGS | 1675200461 | 1665605398 | 99.43% | 50.05 |
| SFY22-13 | Cord blood WGS | 1801444194 | 1782529030 | 98.95% | 56.90 |
| SFY22-14 | Maternal plasma cfDNA deep WGS | 5708651385 | 5661737319 | 99.18% | 108.91 |
| SFY22-14 | Maternal buffy coat WGS | 1409748937 | 1404114857 | 99.60% | 42.88 |
| SFY22-14 | Paternal whole blood WGS | 1518856603 | 1508974610 | 99.35% | 45.44 |
| SFY22-14 | Cord blood WGS | 1081954104 | 1070593586 | 98.95% | 34.20 |
| SFY22-15 | Maternal plasma cfDNA deep WGS | 5855827122 | 5820106577 | 99.39% | 121.78 |
| SFY22-15 | Maternal buffy coat WGS | 1435335596 | 1430547189 | 99.67% | 43.85 |
| SFY22-15 | Paternal whole blood WGS | 1562299445 | 1556098303 | 99.60% | 47.68 |
| SFY22-15 | Cord blood WGS | 1113456219 | 1101319546 | 98.91% | 35.14 |
| SFY22-18 | Maternal plasma cfDNA deep WGS | 6731425030 | 6695075335 | 99.46% | 138.90 |
| SFY22-18 | Maternal buffy coat WGS | 2111539126 | 2060376973 | 97.58% | 63.27 |
| SFY22-18 | Paternal whole blood WGS | 1606403854 | 1554394983 | 96.76% | 47.47 |
| SFY22-18 | Amniotic fluid WGS | 1131005882 | 1121166131 | 99.13% | 35.00 |
| SFY22-19 | Maternal plasma cfDNA deep WGS | 7571893966 | 7530248549 | 99.45% | 149.23 |
| SFY22-19 | Maternal buffy coat WGS | 1243014669 | 1217463515 | 97.94% | 36.45 |
| SFY22-19 | Paternal whole blood WGS | 1610092205 | 1601234958 | 99.45% | 48.37 |
| SFY22-19 | Cord blood WGS | 1307517188 | 1294311264 | 98.99% | 41.24 |
| SFY22-20 | Maternal plasma cfDNA deep WGS | 6484576399 | 6429457500 | 99.15% | 139.70 |
| SFY22-20 | Maternal buffy coat WGS | 2438229495 | 2380118698 | 97.62% | 73.31 |
| SFY22-20 | Paternal whole blood WGS | 2217246772 | 2161148239 | 97.47% | 66.65 |
| SFY22-20 | Cord blood WGS | 478010228 | 468354421 | 97.98% | 14.74 |

Table S3. 56 parameters that were used for neural network model design and training using cord blood WGS data.

| Feature Number | Feature Name | Feature Information | Format |
| --- | --- | --- | --- |
| 1 | index | chromosome and location | chr_N_n |
| 2 | F_A1 | Parental Allele 1 Base is A | 0/1 |
| 3 | F_T1 | Parental Allele 1 Base is T | 0/1 |
| 4 | F_C1 | Parental Allele 1 Base is C | 0/1 |
| 5 | F_G1 | Parental Allele 1 Base is G | 0/1 |
| 6 | F_A2 | Parental Allele 2 Base is A | 0/1 |
| 7 | F_T2 | Parental Allele 2 Base is T | 0/1 |
| 8 | F_C2 | Parental Allele 2 Base is C | 0/1 |
| 9 | F_G2 | Parental Allele 2 Base is G | 0/1 |
| 10 | F_homo | Parental is Homozygous | 0/1 |
| 11 | F_QUAL | Parental Sequencing Overall Quality | positive integer |
| 12 | M_A1 | Maternal Allele 1 Base is A | 0/1 |
| 13 | M_T1 | Maternal Allele 1 Base is T | 0/1 |
| 14 | M_C1 | Maternal Allele 1 Base is C | 0/1 |
| 15 | M_G1 | Maternal Allele 1 Base is G | 0/1 |
| 16 | M_A2 | Maternal Allele 2 Base is A | 0/1 |
| 17 | M_T2 | Maternal Allele 2 Base is T | 0/1 |
| 18 | M_C2 | Maternal Allele 2 Base is C | 0/1 |
| 19 | M_G2 | Maternal Allele 2 Base is G | 0/1 |
| 20 | M_homo | Maternal is Homozygous | 0/1 |
| 21 | M_QUAL | Maternal Sequencing Overall Quality | positive integer |
| 22 | Mc_A1 | Maternal cfDNA Allele 1 Base is A | 0/1 |
| 23 | M_T1 | Maternal cfDNA Allele 1 Base is T | 0/1 |
| 24 | Mc_C1 | Maternal cfDNA Allele 1 Base is C | 0/1 |
| 25 | Mc_G1 | Maternal cfDNA Allele 1 Base is G | 0/1 |
| 26 | Mc_A2 | Maternal cfDNA Allele 2 Base is A | 0/1 |
| 27 | Mc_T2 | Maternal cfDNA Allele 2 Base is T | 0/1 |
| 28 | Mc_C2 | Maternal cfDNA Allele 2 Base is C | 0/1 |
| 29 | Mc_G2 | Maternal cfDNA Allele 2 Base is G | 0/1 |
| 30 | Mc_homo | Maternal cfDNA is Homozygous | 0/1 |
| 31 | Mc_QUAL | Maternal cfDNA Sequencing Overall Quality | positive integer |
| 32 | F_BaseQRankSum |  | 0/1 |
| 33 | F_ExcessHet |  | 0/1 |
| 34 | F_MLEAC |  | 0/1 |
| 35 | F_MQ |  | 0/1 |
| 36 | F_MQRankSum |  | 0/1 |
| 37 | F_QD |  | 0/1 |
| 38 | F_SOR |  | 0/1 |
| 39 | M_BaseQRankSum |  | 0/1 |
| 40 | M_DP |  | 0/1 |
| 41 | M_ExcessHet |  | 0/1 |
| 42 | M_FS |  | 0/1 |
| 43 | M_MLEAC |  | 0/1 |
| 44 | M_MQ |  | 0/1 |
| 45 | M_MQRankSum |  | 0/1 |
| 46 | M_QD |  | 0/1 |
| 47 | M_ReadPosRankSum |  | 0/1 |
| 48 | Mc_BaseQRankSum |  | 0/1 |
| 49 | Mc_ExcessHet |  | 0/1 |
| 50 | Mc_FS |  | 0/1 |
| 51 | Mc_MLEAC |  | 0/1 |
| 52 | Mc_MQ |  | 0/1 |
| 53 | Mc_MQRankSum |  | 0/1 |
| 54 | Mc_QD |  | 0/1 |
| 55 | Mc_ReadPosRankSum |  | 0/1 |
| 56 | Mc_SOR |  | 0/1 |

Table S4. The precision of deduced fetal genotypes confirmed using cord blood or amniotic fluid WGS

| Case No. | All site | | Both heterozygous (ABAB) | | Mother heterozygous (ABAA) | | Father heterozygous (AAAB) | |
| --- | --- | --- | --- | --- | --- | --- | --- | --- |
|  | Number of loci | Consistency rates | Number of loci | Consistency rates | Number of loci | Consistency rates | Number of loci | Consistency rates |
| SFY22-2 | 2125425 | 84.58 | 652351 | 77.88 | 1023654 | 83.71 | 449420 | 96.30 |
| SFY22-6 | 1917654 | 80.33 | 660471 | 74.14 | 868649 | 80.14 | 388534 | 91.26 |
| SFY22-8 | 1698542 | 86.72 | 652354 | 80.98 | 551254 | 83.20 | 494934 | 98.24 |
| SFY22-9 | 1974844 | 84.42 | 624329 | 77.17 | 869013 | 83.51 | 481502 | 95.45 |
| SFY22-13 | 2684512 | 89.02 | 895421 | 82.16 | 1076635 | 88.63 | 712456 | 98.26 |
| SFY22-14 | 2456845 | 86.94 | 1054265 | 81.10 | 831584 | 86.51 | 570996 | 98.35 |
| SFY22-15 | 1925484 | 82.82 | 886542 | 77.65 | 654215 | 80.56 | 384727 | 98.61 |
| SFY22-18 | 2125485 | 87.35 | 706542 | 79.81 | 956521 | 88.67 | 462422 | 96.17 |
| SFY22-19 | 2258745 | 82.50 | 865427 | 71.34 | 895423 | 85.28 | 497895 | 96.90 |
| SFY22-20 | 1834160 | 77.74 | 598336 | 71.47 | 846713 | 77.25 | 389111 | 88.46 |

***Both heterozygous***: maternal heterozygous and paternal heterozygous locus; ***Mother heterozygous:*** maternal heterozygous and paternal homozygous locus; ***Father heterozygous:*** paternal heterozygous and maternal homozygous locus

Table S5. Clinically relevant single nucleotide variants identiﬁed by Self-Learning Neural Network Clinical interpretation of pathogenic/likely pathogenic SNVs determined that was identified high risks and clinical confirmation using WES.

| Case No | Gene Names | Chr:Pos | Transcript | Genotypes | Types of Genetic Variants | Zygote types | Inheritance | Source of Variation | ACMG Classification | Clinical confirmation using WES |
| --- | --- | --- | --- | --- | --- | --- | --- | --- | --- | --- |
| SFY22-2 | FRAS1 | 4:79443902 | NM_025074 | c.10748G>A(p.W3583*) | nonsense | CH | AR | Father | C4: LP | YES |
| SFY22-2 | FRAS1 | 4:79296891 | NM_025074 | c.3152-2A>G() | splice site | CH | AR | Mother | C4: LP | YES |
| SFY22-6 | TOE1 | 1:45805880 | NM_025077 | c.-45G>A | UTR5 | Het | AR | Unknown | C1: B | NO* |
| SFY22-6 | SLC25A20 | 3:48921567 | NM_000387 | c.199-10T>G | splice site | Hom | AR | Both Parents | C5: P | YES |
| SFY22-8 | PKHD1 | 6:51913356 | NM_138694 | c.2341C>T | nonsense | Hom | AR | Both Parents | C5: P | YES |
| SFY22-9 | SCN5A | 3:38590422 | NM_001160160 | c.*1390A>G | UTR3 | Het | AD/AR | Unknown | C2: LB | YES* |
| SFY22-9 | CFAP58 | 10:106163539 | NM_001008723 | c.2092C>T(R698*) | nonsense | Het | AR | Mother | C5: P | YES |
| SFY22-9 | OSGEP | 14:20916116 | NM_017807 | c.740G>A(R247Q) | missense | Hom | AR | Both Parents | C5: P | YES |
| SFY22-9 | TGM6 | 20:2398069 | NM_001254734 | c.1528G>C(D510H) | missense | Het | AD | Father | C3: VUS | YES |
| SFY22-9 | C1GALT1C1 | X:119760629 | NM_001011551 | c.393T>A(D131E) | missense | Hom | XLR | Mother | C2: LB | YES |
| SFY22-13 | FRAS1 | 4:79204019 | NM_025074 | c.1153C>T(p.R385*) | nonsense | CH | AR | Father | C5: P | YES |
| SFY22-13 | FRAS1 | 4:79321972 | NM_025074 | c.4060C>T(p.Q1354*) | nonsense | CH | AR | Mother | C5: P | YES |
| SFY22-14 | FLG | 1:152282818 | NM_002016 | c.4544C>A(S1515*) | nonsense | Het | AD/AR | Mother | C5: P | YES |
| SFY22-14 | SOD3 | 4:24801834 | NM_003102 | c.691C>G(R231G) | missense | Het | Unknown | Father | C1: B | YES |
| SFY22-14 | TH | 11:2189844 | NM_000360 | c.364C>T(R122*) | nonsense | Het | AR | Father | C5: P | YES |
| SFY22-14 | MID1 | X:10437773 | NM_000381 | c.1249C>T(p.Q417*) | nonsense | Hem | XLR | Mother | C5: P | YES |
| SFY22-15 | DSG4 | 18:28970675 | NM_001134453 | c.574T>C(S192P) | missense | Het | AR | Mother | C5: P | YES |
| SFY22-15 | L1CAM | X:153134975 | NM_001143963 | c.1252C>T(Q418*) | nonsense | Hems | XLR | Mother | C5: P | YES |
| SFY22-18 | COL4A1 | 13:110831699 | NM_001845 | c.2263G>A(G755R) | missense | Het | AD | Mother | C5: P | YES |
| SFY22-19 | AGBL1 | 15:87531320 | NM_152336 | c.3323+1G>A | splice site | Het | AD | Mother | C5: P | YES |
| SFY22-19 | HNF1B | 17:36099434 | NM_000458 | c.541C>T(R181*) | nonsense | Het | AD | Mother | C5: P | YES |
| SFY22-20 | KLKB1 | 4:187158034 | NM_000892 | c.428G>A(S143N) | missense | Hom | AR | Both Parents | C1: B | YES |
| SFY22-20 | CCDC170 | 6:151936677 | NM_025059 | c.1810G>A(V604I) | missense | Het | Unknown | Father | C1: B | YES |
| SFY22-20 | GATA4 | 8:11617240 | NM_001308094 | c.*1256A>T | UTR3 | Het | AD | Unknown | C1: B | YES* |
| SFY22-20 | DPYS | 8:105393518 | NM_001385 | c.1468C>T(R490C) | missense | Het | AR | Mother | C5: P | YES |

^*^ ***Clinical confirmation of disease SNVs using fetal cord blood WGS.*** CH-Compound Heterozygous; Hom-Homozygous; Het-Heterozygous; Hem-Hemizygous; P-Pathogenic; LP- Likely Pathogenic.

Table S6. Clinically relevant single nucleotide variants identiﬁed by Self-Learning Neural Network

| Case No. | SFY22-2 | SFY22-2 | SFY22-6 | SFY22-8 | SFY22-9 | SFY22-13 | SFY22-13 | SFY22-14 | SFY22-15 | SFY22-18 | SFY22-19 | SFY22-20 |
| --- | --- | --- | --- | --- | --- | --- | --- | --- | --- | --- | --- | --- |
| Chr:Pos | 4:79443902 | 4:79296891 | 3:48921567 | 6:51913356 | 14:20916116 | 4:79204019 | 4:79321972 | X:10437773 | X:153134975 | 13:110831699 | 17:36099434 | 3: 30686237 |
| Ref/Alt | G>A | A>G | T>G | C>T | G>A | C>T | C>T | C>T | C>T | G>A | C>T | A>G |
| Gene Names | FRAS1 | FRAS1 | SLC25A20 | PKHD1 | OSGEP | FRAS1 | FRAS1 | MID1 | L1CAM | COL4A1 | HNF1B | TGFBR2 |
| HGVS c., p. | NM_025074 | NM_025074 | NM_000387 | NM_138694 | NM_017807 | NM_025074 | NM_025074 | NM_000381 | NM_001143963 | NM_001845 | NM_000458 | NM_003242.6 |
|  | c.10748G>A(p.W3583*) | c.3152-2A>G() | c.199-10T>G | c.2341C>T (p.Arg781*) | c.740G>A(p.Arg247Gln) | c.1153C>T(p.R385*) | c.4060C>T(p.Q1354*) | c.1249C>T(p.Q417*) | c.1252C>T(Q418*) | c.2263G>A(G755R) | c.541C>T (p.Arg181*) | c.95-2A>G |
| Zygotic Types | CH | CH | HO | Hom | Hom | CH | CH | Hem | Hem | Het | Het | Het |
| Inheritance Pattern | AR | AR | AR | AR | AR | AR | AR | XLR | XLR | AD | AD | AD |
| Heterozygous | Father | Mother | Both Parents | Both Parents | Both Parents | Father | Mother | Mother | Mother | Mother | Mother | Father |
| Sequence Ontology | Nonsense | Splice site | Splice site | Nonsense | Missense | Nonsense | Nonsense | Nonsense | Nonsense | Missense | Nonsense | Splice site |
| Classification | C4: LP | C4: LP | C5: P | C4: LP | C4: LP | C4: LP | C4: LP | C5: P | C5: P | C5: P | C5: P | C5: P |
| FF, % | 9.9 | 9.9 | 5.3 | 9 | 14.7 | 9.5 | 9.5 | 7.2 | 4.2 | 14.7 | 12.3 | 2.6 |
| Depth of Plasma cfDNA Average | 124.18 | 124.18 | 113.75 | 71.37 | 101.18 | 97.4 | 97.4 | 108.91 | 121.78 | 138.9 | 149.23 | 139.7 |
| Confirmed by invasive WES | TRUE | TRUE | TRUE | TRUE | TRUE | TRUE | TRUE | TRUE | TRUE | TRUE | TRUE | FALSE |
| Conditions | Fraser syndrome 1 | Fraser syndrome 1 | CACT | PKD4 | GAMOS3 | Fraser syndrome 1 | Fraser syndrome 1 | Opitz GBBB Syndrome | HSAS; MASA; SPG1 | HANAC syndrome; CM-Ⅰ; | RCAD;T2DM | LDS2 |
| OMIM ID | 219000 | 219000 | 212138 | 263200 | 610107 | 219000 | 219000 | 300552 | 308840 | 120130 | 189907 | 190182 |

CH-Compound Heterozygous; Hom-Homozygous; Het-Heterozygous; Hem-Hemizygous; P-Pathogenic; LP- Likely Pathogenic; Carnitine-acylcarnitine translocase (CACT); GAMOS3- Galloway–Mowat syndrome type 3; HSAS-X-linked stenosis of the aqueduct of Sylvius; MASA-mental retardation aphasia, shuffling gait and adducted thumbs; SPG1 -X-linked recessive spastic paraplegia type 1; HANAC-Hereditary angiopathy with nephropathy, aneurysms and muscle cramps syndrome; CM-Ⅰ-Chiari malformation type I; RCAD-Renal cysts and diabetes syndrome; T2DM- Type 2 diabetes mellitus; LDS2-Loeys-Dietz Syndrome Type2; PKD4- polycystic kidney disease-4 with or without polycystic liver disease.

References

1.Miceikaite I. et al. Comprehensive prenatal diagnostics: Exome versus genome sequencing. Prenatal

Diagnosis 2023;43(9): 1132-1141.

2.Richards S. et al. Standards and Guidelines for the Interpretation of Sequence Variants: A Joint Consensus Recommendation of the American College of Medical Genetics and Genomics and the Association for Molecular Pathology. Genet Med 2015;17:405–424.

3.Raman L, Dheedene A, De Smet M, Van Dorpe J, Menten B. WisecondorX: improved copy number

detection for routine shallow whole-genome sequencing. Nucleic Acids Res 2019;47:1605–1614.
