# Supplementary material for: Self-learning neural network as a prediction model in non-invasive prenatal testing to detect fetal SNVs
Source: J Transl Med. 2024 Jul 30;22:707. doi: 10.1186/s12967-024-05433-y (PMC11290223; doi:10.1186/s12967-024-05433-y)
Supplement: Supplementary file 2 — Supplementary Material 2 [file 12967_2024_5433_MOESM2_ESM.pdf]

广东省妇幼保健院医学伦理委员会审查意见书

<table>
<tr><td>项目名称</td><td colspan="5">多种类型遗传疾病同步式无创产前筛查的推广和临床有效性评估</td></tr>
<tr><td>编　号</td><td colspan="5">广东省妇幼保健院医伦第[202301382]号</td></tr>
<tr><td>负责人</td><td>尹爱华</td><td>所在科室</td><td>医学遗传中心</td><td>项目分类</td><td>十四五国家重点研发计划</td></tr>
<tr><td colspan="6">伦理委员会决议:

伦理委员会对医学遗传中心尹爱华递交的“多种类型遗传疾病同步式无创产前筛查的推广和临床有效性评估”十四五国家重点研发计划进行伦理审查。委员们认为其研究内容和方法符合医学伦理规范和要求，同意该项目在我院开展。

主任/副主任委员签名：
2023 年 11 月 28 日</td></tr>
</table>
